# BMP4 Enhances Foam Cell Formation by BMPR-2/Smad1/5/8 Signaling

**DOI:** 10.3390/ijms15045536

**Published:** 2014-03-31

**Authors:** Jun Feng, Jiangfei Gao, Yuxin Li, Yanhua Yang, Lili Dang, Yuanpeng Ye, Jingyuan Deng, Antai Li

**Affiliations:** 1 Department of Cerebral Vessels, First Affiliated Hospital of Medical College, Xi’an Jiaotong University, Xi’an 710061, Shaanxi, China; E-Mail: yuanpeng_ye@163.com; 2 Department of Neurology, Shangluo Central Hospital, Shangluo 726000, Shaanxi, China; E-Mail: jiangfei_gao@163.com; 3 Department of Neurology, the Second Affiliated Hospital, Xi’an Medical College, Xi’an 710038, Shaanxi, China; E-Mail: yuxin_lisx@163.com; 4 Department of Neurology, Shaanxi Armed Police Corps Hospital, Xi’an 710054, Shaanxi, China; E-Mail: yanhua_yangxa@163.com; 5 Department of Neurology, Xingyuan Hospital, Yulin 719000, Shaanxi, China; E-Mail: lili_dang@163.com; 6 Department of Rehabilitation Medicine, First Affiliated Hospital of Medical College, Xi’an Jiaotong University, Xi’an 710061, Shaanxi, China; E-Mail: jingyuan_deng@163.com; 7 Department of Neurology, Xi’an Central Hospital, Xi’an 710003, Shaanxi, China; E-Mail: antai_li@126.com

**Keywords:** foam cell formation, BMP4, BMPR-2, Smad1/5/8

## Abstract

Atherosclerosis and its complications are characterized by lipid-laden foam cell formation. Recently, an obvious up-regulation of BMP4 was observed in atherosclerotic plaque, however, its function and the underlying mechanism remains unknown. In our study, BMP4 pretreatment induced macrophage foam cell formation. Furthermore, a dramatic increase in the ratio of cholesteryl ester (CE) to total cholesterol (TC) was observed in BMP4-treated macrophages, accompanied by the reduction of cholesterol outflow. Importantly, BMP4 stimulation inhibited the expression levels of the two most important cellular cholesterol transporters ABCA1 and ABCG1, indicating that BMP4 may induce formation of foam cells by attenuating transporters expression. Further mechanism analysis showed that BMPR-2, one of the BMP4 receptors, was significantly increased in BMP4 treated macrophage foam cells. That blocking its expression using specific siRNA significantly increased ABCA1 and ABCG1 levels. Additionally, BMP4 treatment triggered the activation of Smad1/5/8 pathway by BMPR-2 signaling. After blocking the Smad1/5/8 with its inhibitor, ABCA1 and ABCG1 expression levels were up-regulated significantly, suggesting that BMP4 inhibited the expression of ABCA1 and ABCG1 through the BMPR-2/Smad1/2/8 signaling pathway. Therefore, our results will provide a new insight about how BMP4 accelerate the progressio of atherosclerosis, and it may become a potential target against atherosclerosis and its complications.

## 1. Introduction

Atherosclerosis is a chronic process resulting in clinically manifest coronary artery disease in middle age and later [1]. Atherosclerosis and its complications like stroke, myocardial infarction, unstable angina, and sudden death, are the leading cause of death and represent a great threat to health [2]. Recently, plaque disruption in atherosclerosis was found to carry the main risk for myocardial infarction, unstable angina, and sudden death [3]. Therefore, a potential direction for cardiovascular diseases is control of vulnerable plaque rupture.

Atherosclerotic plaque is characterized by a large lipid core, thin fibrous cap, and severe inflammation reaction [4]. The large lipid core is the critical component of a vulnerable plaque as lipid-rich and soft plaques are more dangerous than collagen-rich and hard plaques, and are highly thrombogenic after disruption [5]. Studies show that the stability of a vulnerable plaque is significantly increased by decreasing the area of the plaque lipid core, indicating the crucial function of the lipid core in regulating plaque disruption [6,7]. The lipid core is mainly composed of macrophage foam cells, which are found to be the hallmark of early atherosclerosis [8]. Lipid-laden foam cells are formed after phagocytosis of oxidized LDL (ox-LDL) by macrophage cells. They confer one of the highest cardiovascular risks and initiate atherosclerotic progression. Aggressive lowering of lipid levels markedly reduces atherosclerotic coronary lesion, and increases the stability of vulnerable plaque. It has been demonstrated that accelerating the out flux of free cholesterol in macrophages would significantly reduce lipid accumulation. ABCA1 and ABCG1 are two known common regulators of intracellular cholesterol outflow that play an important role in regulating foam cell formation. ABCA1 and ABCG1 transport free cholesterol from within the cell to lipid-poor apoA1 particles to generate HDL, a function critical for the efflux of excess cholesterol from cells in peripheral tissues [9–11].

Bone morphogenetic proteins (BMPs), members of the transforming growth factor β (TGF-β) superfamily, are multi-functional growth factors that play key roles in embryogenesis, skeletal formation, hematopoiesis, and neurogenesis. In recent years, the role of BMP in atherosclerosis, sclerosis, and their complications has attracted great attention. Research shows that inhibition of BMP signaling increases lipid efflux, thereby reducing foam cell formation, intracellular lipid accumulation, and atherosclerosis [12,13]. BMP4 is one of the key members in the BMP family, mainly functioning in skeletal repair, angiogenesis, and kidney formation [14–16]. Recently, BMP4 was found to be overexpressed in the atherosclerotic plaque, and was found to be crucial in cellular functions including vascular calcification, inflammation, and smooth muscle cell proliferation [17,18]. However, its role in atherosclerosis and the underlying mechanism remain unclear.

In this study, we explored the effect of BMP-4 on foam cell formation. To further analyze the mechanism underlying this process, expression levels of ABCA1 and ABCG1 were assessed. Furthermore, BMP receptor 2 (BMPR-2)/Smad1/5/8 signaling was also evaluated.

## 2. Results and Discussion

### 2.1. BMP4 Facilitated Foam Cell Formation

**Figure 1.**
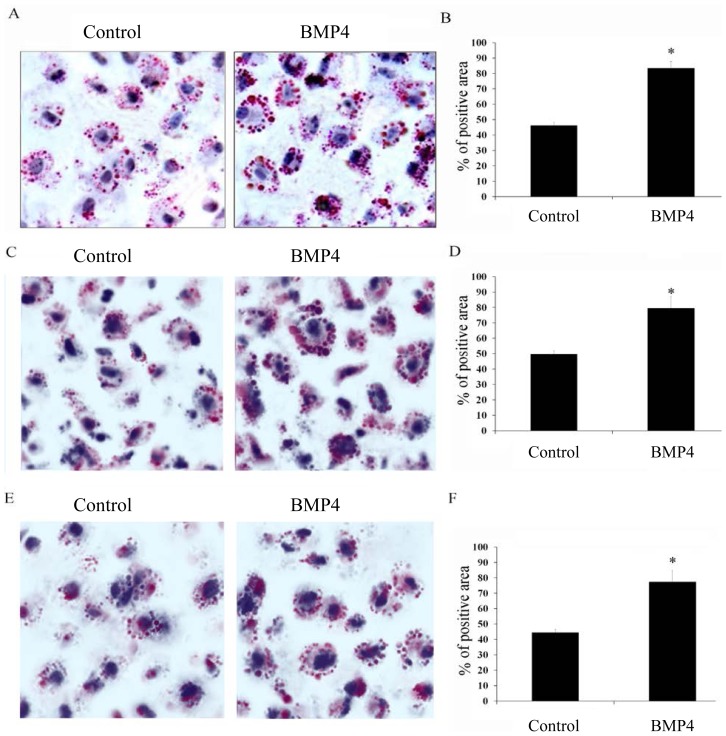
Effect of BMP4 on macrophage foam cell formation. After pretreating with PBS (control) or BMP4 for 48 h, cells were stained with Oil Red O. 400× photomicrographs and the quantitative analysis of the RAW 246.7 cells (**A**) and (**B**), MPM (**C**) and (**D**) and PBMCs (**E**) and (**F**) were demonstrated equivalent macrophage area density but higher percent of Oil Red O positive area compared with controls. * *p* < 0.05.

To evaluate the impact of BMP4 on foam cell formation, RAW 246.7 cells, MPM and PBMCs were co-incubated with ox-LDL, individually, with pretreatment of BMP4 or not (control). Compared with the control group, an obvious increase in RAW 246.7 cells foam cell formation was observed in BMP4-treated groups by Oil Red O staining (Figure 1A,B). The same increasing in Oil Red O positive area were observed in cells of MPM (Figure 1C,D) and PBMCs (Figure 1E,F), treated with BMP4. Taken together, our results suggested that BMP-4 could enhance foam cell formation.

### 2.2. Effect of BMP4 on Lipid Deposition

**Figure 2.**
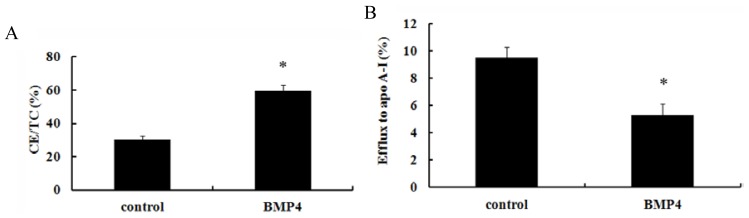
Effect of BMP4 to cellular lipid. Cellular lipid was extracted, and free cholesterol (FC) and total cholesterol (TC) were analyzed by HPLC. CE and the CE/TC ratio were calculated. (**A**) CE:TC ratios in macrophage cells after fatty acid treatments with or without BMP4 (4 ng/L) for 48 h; (**B**) cholesterol efflux in macrophage cells after fatty acid treatments with or without BMP4 (4 ng/L) for 48 h. * *p* < 0.05.

It is known that lipid accumulation triggers foam cell formation, which can be attenuated by enhancing cholesterol efflux [19,20]. To further analyze the effect of BMP4 on lipid deposition, the CE/TC ratio and cholesterol efflux were evaluated. HPLC analysis showed that the CE/TC ratio was 59.68% in the BMP4-induced group, which was about twofold higher than that of controls (30.21%), suggesting that BMP4 transfection increased lipid accumulation in murine macrophage foam cells (Figure 2A). To ascertain whether BMP4 could induce lipid outflow in macrophages, ox-LDL was added and the ratio of efflux to apoA1 was used to analyze cholesterol outflow. As shown in Figure 2B, cholesterol outflow was 5.29% in the BMP4-induced group, which was significantly lower than that of control (9.50%), indicating an inhibitory effect of BMP4 on foam cell formation and lipid accumulation (Figure 2B). Accordingly, it can be concluded that BMP4 promotes the formation of foam cells by inhibiting cholesterol outflow.

### 2.3. BMP4 Inhibits ABCA1 and ABCG1 Expression

**Figure 3.**
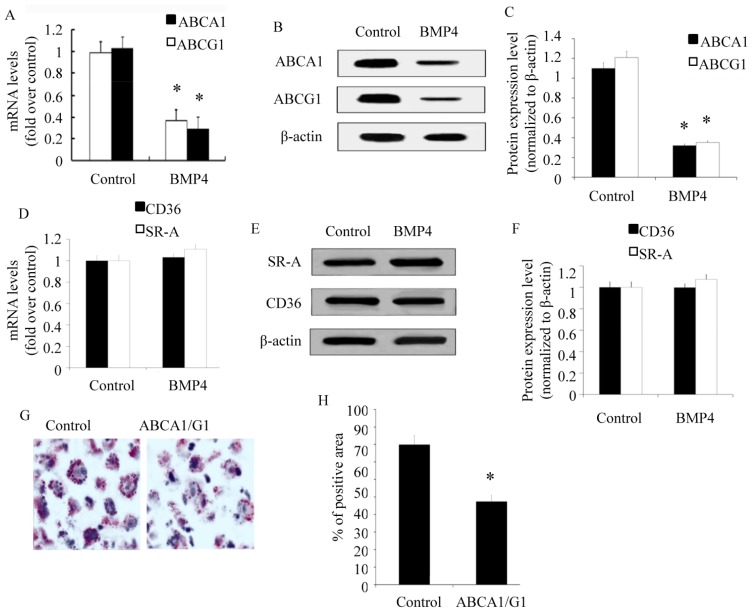
BMP4 inhibits ABCA1 and ABCG1 expression level. After treating with PBS (control) or BMP4, the expression levels of ABCA1 and ABCG1 were evaluated using RT-PCR (**A**) and Western blots (**B**), and the Western blots were quantified with relative densitometry to β-actin (**C**). Simultaneously, the expression levels of SR-A and CD36 were evaluated using RT-PCR (**D**) and Western blots (**E**), and the Western blots were quantified with relative densitometry to β-actin (**F**). After treating with BMP4, macrophages with null vector transfection (control) or ABCA1 and ABCG1 over-expression vector transfection, the foam cell formation of macrophages were detected using Oil red O (**G**), and was quantified with Oil red O positive area relative to macrophage area (**H**). * *p* < 0.05.

The ABC transporter family, especially ABCA1 and ABCG1, is primarily involved in the efflux of cholesterol from macrophages and subsequent foam cell formation [21]. ABCA1 and ABCG1 can transport free cholesterol to lipid-poor apoA1 particles to generate HDL, a function that is critical for the efflux of excess cholesterol from cells in peripheral tissues [9]. To investigate the mechanism underlying the effect of BMP4 on foam cell formation, expression of ABCA1 and ABCG1 were evaluated. RT-PCR analysis indicated that the mRNA levels of ABCA1 and ABCG1 was respectively 0.37-fold and 0.29-fold higher after BMP4 treatment in comparison with controls, implying a significant down-regulation of ABCA1 and ABCG1 mRNAs in BMP4-induced groups (Figure 3A). Western blotting analysis showed a significant reduction in protein levels (Figure 3B,C). Therefore, our results proved that BMP4 negatively modulated the expression of ABCA1 and ABCG1, which would accelerate foam cell formation by inhibition of ABC transporters. The type A scavenger receptor (SR-A) and CD36 were two of the macrophage scavenger receptors (SRs), and were implicated in processes that contribute to early foam cell formation and further to the progression toward more complex vulnerable plaques. In this study, we further analyzed the expression of SR-A and CD36 using RT-PCR and western blotting analysis. There was no difference in the expression of CD36 pretreated with BMP4 or not, whiles the expression of SR-A was somewhat elevated, but no significant statistical difference.

To further confirm the conclusion that BMP4 inhibited the formation of foam cells by decreasing ABCA1 and ABCG1 expression, macrophages with overexpression of ABCA1 and ABCG1 were established. After being pretreated with BMP4, Oil red O analysis detected an decreased posit area in macrophages with overexpression of ABCA1 and ABCG1 compared with control, indicating that the overexpression of ABCA1 and ABCG1 could mostly rescue the BMP4-dependent up-regulatory effect on foam cell formation.

### 2.4. BMPR-2 Is Responsible for BMP4-Inhibited ABCA1 and ABCG1 Expression

**Figure 4.**
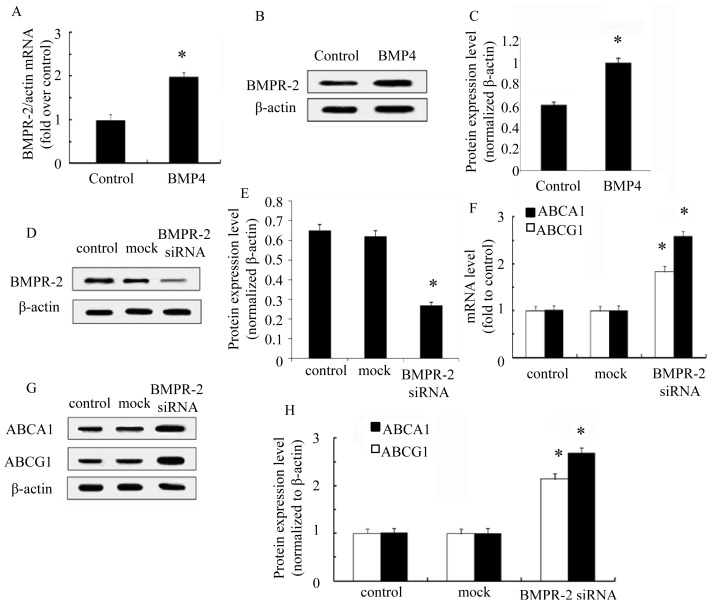
BMPR-2 is responsible for BMP4 inhibition of ABCA1 and ABCG1 expression. Unless otherwise specified, all macrophages below were cultured in DMEM with oxLDL. After treatment with PBS (control) or BMP4, macrophages were cultured for 48 h. Then the BMPR-2 expression was detected using RT-PCR (**A**), Western blots (**B**) and its quantification with relative densitometry of BMPR2/β-actin (**C**). After treatment with PBS (control), mock, or BMPR-2 siRNA, the expression of BMPR-2 in macrophages were evaluated using Western blots (**D**) and its quantification with relative densitometry of BMPR2/β-actin (**E**), then the ABCA1 and ABCG1 expression were using RT-PCR (**F**), Western blots (**G**) and its relative quantification over β-actin (**H**). * *p* < 0.05.

BMPR-2 is a member of the TGF-β superfamily, and is critical for BMP signaling, which is involved in multiple physiological characteristics including many developmental processes [22]. Plenty of research shows that BMPR-2 usually works as the key receptor for BMP4 [23]. Therefore, to further clarify the mechanism underlying BMP4-mediated ABCA1 and ABCG1 expression, the expression level of BMPR-2 was analyzed. As shown in Figure 4A, BMPR-2 mRNA levels were markedly increased in BMP4-treated macrophage foam cells compared with control group. A similar increase in BMPR-2 protein levels was also observed by Western blotting analysis (Figure 4B,C), indicating that BMP4 could induce the expression of BMPR-2. We further analyzed the connection between BMPR-2 and ABC transporters. The expression of BMPR-2 was dramatically silenced by its specific siRNA (Figure 4D,E). After BMPR-2 siRNA transfection, the expression level of ABCA1 was significantly increased (Figure 4F–H). A similar increasing in ABCG1 expression was confirmed, suggesting that BMP4 regulated the expression of ABCA1 and ABCG1 by the BMPR-2 pathway (Figure 4). In summary, the results confirmed that BMP4 regulated the expression of ABCA1 and ABCG1 by the BMPR-2 pathway, suggesting the important role of the BMP/BMPR-2 pathway in expression of ABC transporters to mediate foam cell formation and plaque progression in the area of the atherosclerotic lesion.

### 2.5. BMP4 Induces Foam Cell Formation by BMPR-2/SMAD1/5/8 Signaling

**Figure 5.**
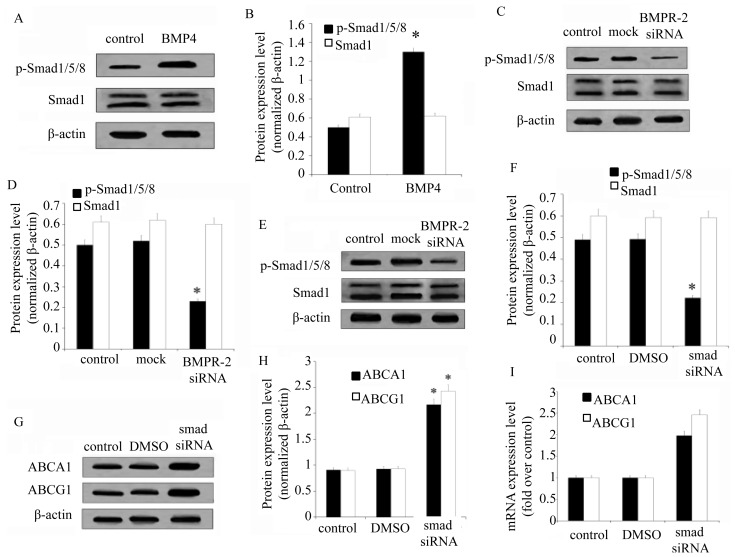
BMP4 induces foam cell formation by BMPR-2/Smad1/5/8 signaling. Unless otherwise specified, macrophages of murine RAW264.7 cell line below were cultured in DMEM with oxLDL. After treatment with PBS (control) or BMP4, macrophages were cultured for 48 h. Then the expression of p-Smad1/5/8 and Smad1 were evaluated using Western blot (**A**) and quantified with relative densitometry over β-actin (**B**). Then, macrophages were treated with PBS (control), mock, or BMPR-2 siRNA, the expression of BMPR-2 in macrophages were evaluated using Western blots (**C**) and quantified with relative densitometry (**D**). In addition that the macrophages were treated with PBS (control), DMSO, or Smad siRNA, p-Smad1/5/8 and Smad1 were evaluated using Western blot (**E**) and quantified with relative densitometry over β-actin (**F**), then the ABCA1 and ABCG1 expression were evaluated using Western blots (**G**) and its relative quantification over β-actin (**H**) and RT-PCR (**I**). * *p* < 0.05.

The Smad signaling pathway is critical for mediating TGF-β superfamily signals from the cell surface to the nucleus, and is functioned as transcriptional co-modulators to regulate TGF-β-dependent gene expression [24,25]. Smad1, 5, and 8 are closely related to BMPR-2 [26]. Studies reported that Smad1/5/8 was responsible for receiving BMPR-2 signals, which in turn initiated intracellular signaling through phosphorylation [19]. To explore the mechanism underlying BMPR-2 regulation of ABC expression in murine macrophage foam cells, the level of Smad1/5/8 phosphorylation was quantified. Western blotting analysis showed that Smad1/5/8 activity was significantly up-regulated after BMP4 preconditioning (Figure 5A). In further investigations to explore whether BMPR-2 induced Smad1/5/8 phosphorylation, siRNA knockdown of BMPR-2 was carried out. The results showed a great inhibition of Smad1/5/8 phosphorylation (Figure 5B). The p-Smad1/5/8 was blocked with its specific siRNA, Smad1/5/8 phosphorylation was decreased (Figure 5E,F), concomitant with a significantly increase in ACBA1 and ABCG1 expression (Figure 5G,H). Consistent with these results, the mRNA levels of ABCA1 and ABCG1 were significantly up-regulated when the Smad1/5/8 pathway was blocked in BMP-4-treated cells (Figure 5I). In conclusion, these results demonstrated that BMP4 induced murine macrophage foam cell formation by ABCA1 and ABCG1 though activation of the BMPR-2/Smad1/5/8 signaling pathway.

### 2.6. Discussion

Atherosclerosis is generally deemed to be a chronic disease caused by uptake of ox-LDL into macrophages [20]. Formation of lipid-laden foam cells is a key step in early atherogenesis [20]. Therefore, lipid lowering was used in current clinical treatment to prevent atherosclerotic progression. An increase in macrophage cholesterol efflux attached close attention, which was thought to be a promising target to prevent atherosclerotic progression. Inhibition of lipid accumulation was found to be an effective measure to improve atherosclerosis and plaque instability [21].

A report has suggested that several BMP-family ligands can increase the process of atherosclerosis [22]. In atherosclerotic lesions, BMP was recently reported to be up-regulated, but the specific function and mechanism have not been clearly understood until now [23]. The present study has found that BMP4 accelerates macrophage foam cell formation, indicating its important role in atherosclerosis. The CE/TC ratio was used as a standard in evaluation of lipid accumulation. The cellular CE/TC ratio was significantly increased by BMP4, together with the inhibition of intracellular cholesterol efflux, indicating that BMP4 may play a key role in maintaining intracellular cholesterol levels within macrophages to accelerate foam cell formation.

ABC genes are essential for many processes in the cell and play an important role in cardiovascular disease [27]. We recently identified the ABC transporters ABCA1 and ABCG1 as the major genes underlying the HDL deficiency associated with reduced cholesterol efflux [27,28]. ABCA1 transports free cholesterol from within the cell to lipid-poor apoA1 particles, playing a critical role for the efflux of excess cholesterol from cells [27]. Acting in concert with ABCA1, ABCG1 is another member of the ABC family that it is responsible for cellular cholesterol efflux to further lipidate HDL, indicating the relevance of the ABC transporter family to the removal of cholesterol from lipid-engorged foam cells that accumulate in atherosclerotic lesions [29]. To explore the mechanism underlying BMP4 induction of foam cell formation, ABCA1 and ABCG1 expression was evaluated using Western blotting and RT-PCR in BMP4-treated cells. A significant decrease in the mRNA and protein levels of ABCA1 and ABCG1 was detected, proving the significant down-modulatory effect of BMP4 on ABCA1 and ABCG1 expression. Furthermore, the overexpression of ABCA1 and ABCG1 could well rescue the BMP4-dependent up-regulatory effect on foam cell formation. Accordingly, we conclude that BMP4 can mediate the outflow of cholesterol by down-regulating the expression of ABCA1 and ABCG1, slowing down the process of atherosclerotic plaques and subsequent plaque instability.

BMPR-2 is the receptor protein that plays a key role in the BMP4 signaling pathway [30,31]. Meanwhile, increasing research has demonstrated that BMPR-2 was closely related to atherosclerosis [32]. Therefore, we investigated in this study the role of BMPR-2 in BMP4-induced macrophage foam cell formation. As expected, BMPR-2 was up-regulated in macrophage foam cells treated with BMP4. Western blotting analysis showed an identical increased expression with RT-PCR in mRNA levels, indicating that the BMPR-2 signaling pathway might be involved in BMP4-induced foam cell formation. Silencing of BMPR-2 using specific siRNA significantly increased ABCA1 and ABCG1 expression, confirming that BMPR-2-mediated transmission of BMP4 signals inhibited ABCA1 and ABCG1 expression and lipid efflux, thereby promoting foam cell formation.

**Figure 6.**
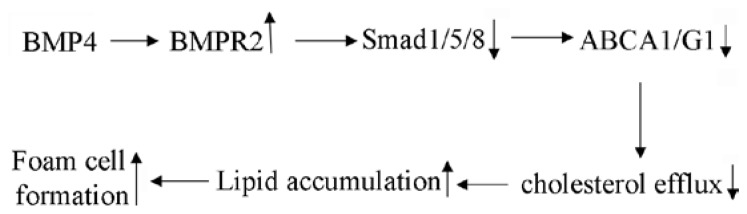
A schematic model presenting the pathway of BMP4 to affect foam cell formation.

Smad1, 5, and 8 are three BMP receptors that regulate gene expression by binding to target gene DNA [33]. The level of Smad1/5/8 activity was quantified in this study to explore the mechanism through which BMPR-2 regulates the expression of ABC in murine macrophage foam cells. Western blotting showed a significant increase in Smad1/5/8 after induction by BMP4. Silencing BMPR-2 by siRNA led to a decrease in Smad1/5/8, showing that Smad1/5/8 activity correlated directly with BMP4 and participated in the foam cell formation of atherosclerosis. Further analysis confirmed that blocking of the Smad1/5/8 pathway with a Smad1/5/8 inhibitor increased the expression of ABCA1 and ABCG1, suggesting that BMP4 induced murine macrophage foam cell formation by inhibiting the expression of ABCA1 and ABCG1 through the BMPR-2/Smad1/2/8 signaling pathway (Figure 6).

In summary, our studies demonstrated that BMP4 induced lipid-laden foam cell formation by inhibiting cholesterol efflux via ABCA1 and ABCG1 through the BMPR-2/Smad1/2/8 signaling pathway. Consequently, BMP4 may become a promising therapeutic agent against atherosclerosis and its complications. Further studies are needed to investigate the function of BMP4 silencing and the underlying molecular mechanisms during the process of plaque instability in the future.

## 3. Experimental Section

### 3.1. Reagents and Antibodies

DMEM medium and fetal bovine serum was purchased from BioWhittaker (Walkersville, MD, USA). Rabbit anti-rat ABCAl and ABCGl polyclonal antibodies were obtained from BioPorto Antibodyshop (Gentofte, Denmark). Synthetic small interfering RNA (siRNA), Oil Red O, and Trizol were purchased from the Invitrogen Company (Life Technologies, Grand Island, NY, USA).

#### Cells

Murine RAW-246.7 macrophages were purchased from the American Type Culture Collection (ATCC) (Manassas, VA, USA). The primary mouse peritoneal macrophages (MPM) and Peripheral blood mononuclear cells, PBMCs, were isolated before. The specific steps were as followed. MPM were harvested from BALB/c mice. The sterile (1 mL 3.85% thioglycollate) were injected into mice for 48 to 72 h. Then, the peritoneal cavity was obtained by washing with 10 mL of RPMI 1640 culture medium containing 2% FBS. At last, the MPM were allowed to adhere for 2 h and washed twice before treatment. This study was approved by the Ethics Committee of the First Affiliated Hospital of Medical College in accordance with the Declaration of Helsinki. To isolate PBMCs, peripheral blood (5–10 mL) was obtained from healthy volunteers in a fasting state in the following morning of the admission day written informed consent. The PBMCs were separated by ficoll density gradient centrifugation and separation, and CD4^+^ T cells were purified by negative selection (RosetteSep; StemCell Technologies, Vancouver, BC, Canada). Th1 cells were isolated by negative selection using anti-CD4^+^CXCR3^+^CCR6^−^ microbeads (Miltenyi Biotec, Teterow, Germany).

### 3.2. LDL Isolation and Oxidization

LDL was isolated from fresh normal human plasma according to the method described previously [16]. In brief, the low-density fraction after isolation of very low-density lipoproteins was aliquoted into Quickseal tubes. The volume and density of the solution in each tube were adjusted to 35 mL and 1.063 kg/L, respectively, with buffer (38% NaBr and 0.15 M NaCl). The tubes were sealed and centrifuged at 15,000× *g* at a temperature of 4 °C for 20 h. Approximately 5 μL LDL (1.063 kg/L) was recovered from the supernatant in each tube and was oxidized by exposure to CuSO_4_ (10 μmol/L) for 20 h at 37 °C, then the Cu^2+^ was removed by extensive dialysis. Increased mobility in agarose gel and an increased level of thiobarbituric acid-reactive substances were used to assess the degree of oxidation of ox-LDL (compared with native LDL).

### 3.3. Cell Culture and Treatment

Cells were grown in DMEM medium supplemented with 10% (*v*/*v*) fetal bovine serum, 100 U/mL penicillin, and 0.1 mg/mL streptomycin at 37 °C in a humidified sterile condition containing 95% air and 5% CO_2_, and split every 2 days by gentle scraping. Then, macrophages were treated with a final concentration of 60 mg/L ox-LDL in six-well plates at 37 °C for 12 h.

### 3.4. Oil Red O Staining

The RAW 246.7 cells, MPM and PBMCs were cultured with oxLDL for 12 h, individually, and then washed three times with PBS. After stimulation of macrophages with oxLDL for 12 h, macrophage foam cell formation was monitored by Oil-Red O staining before or after pretreating with BMP4 for 1 h. Briefly, cells were fixed in 4% frozen formaldehyde for half an hour, followed by washing with PBS. Then, they were fixed in propylene glycol for 5 min, stained for 15 min with 0.5% (*w*/*v*) Oil Red O solution in propanediol, and excess water was evaporated at 60 °C. Cells were then washed for 5 min with 85% propanediol and several times with PBS.

### 3.5. CE/TC Analysis by High-Performance Liquid Chromatography (HPLC)

RAW 246.7 cells were pretreated with BMP4 for 1 h and then incubated for 48 h in the presence of ox-LDL. After incubation, the cells were washed, thrice, with PBS and harvested in PBS. They were sonicated in an ice bath using an ultrasonic processor. The protein concentration of the lysate was then determined using the BCA assay with a portion of the lysate (100 μL) transferred to microfuge tubes. Then, stigmasterol (150 μL) was added to the rest of the lysate (450 μL) and mixed by vortexing. For the measurement of TC and FC, the mix was divided into two equal parts and KOH (100 μL 8.9 mol/L) was added to the TC sample, while the FC sample did not need to be saponified in KOH solution. After 2 h of incubation in a 50 °C water bath, 1 mL hexane was added to all tubes and subsequently centrifuged at 8000× *g* at 4 °C for 15 min. The supernatant of organic phases was collected and dried in a SpeedVac. The residues were collected and re-suspended in 400 μL acetonitrile:isopropanol (80:20, *v*/*v*) for further analysis. Then, the samples was injected in the HPLC system separately and analyzed with a System Chromatographer (PerkinElmer Inc., Waltham, MA, USA). Total Chrom software (PerkinElmer Inc.) was used to analyze the data. At the end, CE values were obtained by subtracting the FC values from the TC values. CE/TC was calculated using the following formula: (TC − FC)/TC × 100%.

### 3.6. Assessment of Cholesterol Efflux

RAW 246.7 cells were treated with [^3^H] cholesterol for 48 h to label cholesterol. To allow for equilibration of [^3^H] cholesterol with intracellular cholesterol, cells were washed and incubated for an additional 24 h in media containing bovine serum albumin (2 g/L). They were then incubated with BFA (4 μmol/L) or various concentrations of HDL3 (50, 100, and 200 mg/L) in serum-free media, which were recovered 24 h later. Cells were dissolved in HEPES (1 mmol/L, pH 7.5) containing 0.5% Triton X-100. Media were centrifuged at 8000× *g* for 3 min to remove nonadherent cells. For the determination of radioactivity, aliquots of both cells and supernatants were then subjected to scintillation (FJ-2107P type liquid scintillator, Xi’an Nuclear Instrument Factory, Xi’an, China). Cholesterol efflux data were obtained using the following formula: [^3^H] cholesterol in medium/([^3^H] cholesterol in cells + [^3^H] cholesterol in medium) × 100%.

### 3.7. Real-Time Reverse Transcription Polymerase Chain Reaction (RT-PCR)

Total RNA was prepared using the Trizol reagent (Invitrogen) and the optical absorbance ratio at 260 nm/280 nm was measured to determine the content. Then, the RNA was reverse transcribed into cDNA with random hexamer primers. Quantitative RT-PCR analysis was performed with cDNA as the template to amplify ABCA1, ABCG1, SR-A, CD36, and BMPR-2 mRNAs with specific primers (Table 1). The reaction was as follows: 10 min 95 °C, then 40 cycles of 1 min 95 °C, 2 min 63 °C, and 1 min 72 °C, and then a final annealing step at 72 °C for 10 min. The mRNAs of ABCA1, ABCG1, and BMPR-2 were normalized to β-Actin (mouse) mRNA with the comparative Ct method. The sequences of primers for ABCA1, ABCG1, and BMPR-2 were set as follows: ABCA1, 5′-TGTCCAGTCCAGTAATGGTTC TGT-3′ and 5′-AAGCGAGATATGGTCCGGATT-3′, ABCG1 primers: 5′-CCAGAAGTCGGA GGCCATC-3′ and 5′-AAGTCCAGGTACAGCTTGGCA-3′, BMPR-2 primers, 5′-GTGCCCTGGCT GCTATGG-3′ and 5′-TGCCGCCTCCATCATGTT-3′, SR-A primers, 5′-CCAGGGACATGGGAA TGCAA-3′ and 5′-CCAGTGGGACCTCGATCTCC-3′, CD36 primers: 5′-TCCAGCCAATGCCTT TGC-3′ and 5′-TGGAGATTACTTTTCAGTGCAGAA-3′.

### 3.8. Western Blotting

RAW 246.7 cells were lysed with 200 μL lysis buffer containing 20 mmol/L HEPES, 25 mmol/L MgCl, 5 mmol/L KCL, 0.5% (*v*/*v*) complete protease inhibitor, and Triton X-100. Then, the debris was removed by centrifugation at 12,000× *g* at 4 °C for 10 min. Equal amounts of cell protein (typically 80 μg) were separated using 8% precast SDS-PAGE gels (Invitrogen) and electrophoretically transferred to PVDF membrane. The membranes were subsequently probed individually with 1:150 polyclonal primary ABCA1 antibody, ABCG1 antibody, SR-A antibody, CD36 antibody, or BMPR-2 antibody (BD Transduction Laboratories, San Jose, CA, USA) or 1:1000 SMAD1/5/8 antibody (Cell Signaling Technology, Beverly, MA, USA). Detection was by incubation with goat anti-mouse immunoglobulin G (IgG; 1:5000; Sigma) followed by enhanced chemiluminescence (ECL, Amersham Pharmacia, NJ, USA). The intensity of the bands was measured using labwords analysis software (Shenteng, Shanghai, China).

#### 3.8.1. Generation of ABCA1/G1 Overexpression Vector ABCA1

The full-length murine (6.9-kb) ABCA1 cDNA and (5.4-kb) ABCG1 were cloned into the expression plasmid pcDNA3.1 vector (Invitrogen) to make the full-length mouse Abca1/g1 cDNA. Then, complete sequencing was used verify the correct sequence and orientation. XhoI was used to excise ABCA1 and ABCG1. The ABCA1 and ABCG1 with cohesive end of XhoI were then cloned into MoPrP.HD-N171 TG mouse vector. In addition, the correct ABCA1 and ABCG1 overexpression vector was verified by complete sequencing. The constructed vector was transfected into RAW 246.7 cells using Lipofectamine™ 2000 (Invitrogen, Carlsbad, CA, USA) to constructed cells overexpression ABCA1 and ABCG1. In addition, a vacant plasmid MoPrP.HD-N171 was transfected into RAW 246.7 cells using Lipofectamine™ 2000 as the control group. All of the cells were used for experiments at 72 h after transfection.

#### 3.8.2. RNA Interference

The siRNA sequences targeting mouse BMPR-2, Smad1, and Smad5 were from Invitrogen; general siRNA is available upon request. The siRNAs were transfected as described [34]. SiRNA sequences for BMPR-2 were synthesis. Macrophage cells were seeded into six-well plates. After 24-h incubation, transfection using Lipofectamine 2000 (Invitrogen) was performed following the manufacturer’s protocols. Twenty-four hours following transfection, BMPR-2 mRNA and protein levels were determined, by RT-PCR and Western blotting, respectively. The reported data are the average of three or four independent experiments.

### 3.9. Statistical Analysis

All the data obtained were analyzed using SPSSl7.0 statistical software. The results are expressed as mean ± standard deviation (SD). Methods including least significant difference *t*-test, Dunnett’s test, and analysis of variance were applied appropriately to evaluate differences between groups. A *p* < 0.05 was considered statistically significant.

## 4. Conclusions

Our study demonstrated that BMP4 could induce macrophage foam cell formation by ABCA1 and ABCG1 expression through the BMPR-2/Smad1/2/8 signaling pathway. Therefore, BMP4 may become a potential agent against atherosclerosis and its complications.
